# Experimental Study of the Vortex-Induced Vibration of Drilling Risers under the Shear Flow with the Same Shear Parameter at the Different Reynolds Numbers

**DOI:** 10.1371/journal.pone.0104806

**Published:** 2014-08-13

**Authors:** Mao Liangjie, Liu Qingyou, Zhou Shouwei

**Affiliations:** 1 State Key Laboratory of Oil and Gas Reservoir Geology and Exploitation, Southwest Petroleum University, Sichuan, Chengdu, China; 2 China National Offshore Oil Corporation, Beijing, China; Tianjin University, China

## Abstract

A considerable number of studies for VIV under the uniform flow have been performed. However, research on VIV under shear flow is scarce. An experiment for VIV under the shear flow with the same shear parameter at the two different Reynolds numbers was conducted in a deep-water offshore basin. Various measurements were obtained by the fiber bragg grating strain sensors. Experimental data were analyzed by modal analysis method. Results show several valuable features. First, the corresponding maximum order mode of the natural frequency for shedding frequency is the maximum dominant vibration mode and multi-modal phenomenon is appeared in VIV under the shear flow, and multi-modal phenomenon is more apparent at the same shear parameter with an increasing Reynolds number under the shear flow effect. Secondly, the riser vibrates at the natural frequency and the dominant vibration frequency increases for the effect of the real-time tension amplitude under the shear flow and the IL vibration frequency is the similar with the CF vibration frequency at the Reynolds number of 1105 in our experimental condition and the IL dominant frequency is twice the CF dominant frequency with an increasing Reynolds number. In addition, the displacement trajectories at the different locations of the riser appear the same shape and the shape is changed at the same shear parameter with an increasing Reynolds number under the shear flow. The diagonal displacement trajectories are observed at the low Reynolds number and the crescent-shaped displacement trajectories appear with an increasing Reynolds number under shear flow in the experiment.

## Introduction

Drilling risers are key equipment to connect drilling platform and subsea blowout-preventer in deep-water drilling. Vortexes are formed beside the drilling risers when the flowing ocean currents over the risers. Drilling risers vibrate both in in-line (IL) and cross-flow (CF) directions for the effect of vortex-induced forces. This phenomenon is called vortex-induced vibration (VIV)[1]-[3]. When the vortex shedding frequencies are close to the natural frequencies of the risers, vortex shedding lock-in occurs. This condition causes significant fatigue of the risers, thereby affecting safety of drilling platform and crews.

A considerable number of experiment studies and numerical simulations[4]-[14] for VIV under the uniform flow have been performed so far. However, currents are not uniform, but sheared. Marine structures like drilling risers are under the effect of shear flow currents. Therefore, investigating VIV mechanism under shear flow is necessary.

Jordan & Fromm[15] investigated the laminar flow over a circle in a shear flow at the Reynolds number of 400. Kiya[16] investigated the effect of shear flow on a circular cylinder by experiment with the shear parameter from 0 to 0.25 and Reynolds number from 35 to 1500, their investigation showed that the critical Reynolds number beyond which vortex shedding from the cylinder occurred was found to be higher than that for a uniform stream. Kwon[17] carried out an experiment to study the shear flow over a circular cylinder with the shear parameter (0<*k*<0.25) and the Reynolds number (600<*Re*<1600) and the experiment results indicated the drag coefficient decreased with increasing *Re*. Balasubramanian & Skop[18] introduced a diffusively coupled Van der Pol oscillator and successfully modeled VIV in linearly sheared flows. Vandiver et al.[2], [19] found that lock-in might occur under highly sheared conditions and explained that the probable reason of the lock-in was the power likely to one particular mode may dominate all other modes under highly sheared conditions. Kurose & Komori[20] studied the drag and lift forces on a rotating sphere in a linear shear flow by means of a three-dimensional numerical simulation. Lei[21] solved two dimensional Navier-Stokes equations and pressure Poisson equation with a finite difference method to investigate VIV under the shear flow and showed that the Strouhal number and the drag coefficient decreased as the shear parameter increased. Kang[22] investigated two-dimensional laminar flow over a circular cylinder under the effect of a uniform planar shear at low Reynolds numbers by numerical method and his studies showed that shedding frequency and the mean drag remained constant or slightly decreased with increasing shear rate. Sumner & Akosile[23] conducted an experiment to investigate the effect of the uniform planar shear flow on a circular cylinder at subcritical Reynolds number and their experiment results indicated that low to moderate shear caused a small increase in the Strouhal number, an increase in the mean base pressure coefficient, a reduction in the mean drag force coefficient. Marcollo & Hinwood[24] designed a new experimental facility to research the CF and IL lock-in mechanisms under the sheared flow and showed that the motion of the cylinder in a sheared flow might display single or multi-mode behavior depending on the power input to each of the competing modes. Lie & Kaasen[25] conducted an experiment to study large-scale model in linearly sheared flow and depicted the information of the VIV with high order of responding modes. Huang et al.[26] studied a vertical riser VIV under sheared current by using numerical method and made a comparison with published experimental data. Lin & Wang[27] found that the shear parameter had a significant effect on the response of risers by solving the Morison equation and wake oscillator model. Despite these studies, experiment research on VIV with the effect of shear flow currents of drilling riser is scarce.

This study aims to investigate the effect of shear flow on VIV of the drilling risers with the same shear parameter at the two different Reynolds numbers. In order to investigate VIV mechanisms more thoroughly, we carried out the experiment in a deepwater offshore basin in State Key Laboratory of Ocean Engineering in Shanghai Jiao Tong University. The instrumented drilling riser was 8 m long and made of PVC. The riser was towed vertically in a deepwater offshore basin under the effect of shear flow current generated from the current generation system. Various measurements were obtained by the fiber bragg grating (FBG) strain sensors placed on the riser, and VIV under the shear flow with the same shear parameter at the different Reynolds number was investigated.

## Experiment

### Ethics Statement

The experiment was conducted in a deep-water offshore basin at the State Key Laboratory of Ocean Engineering in Shanghai Jiao Tong University. Southwest Petroleum University and China National Offshore Oil Corporation designed and carried out the experiment, and paid fee to use a deep-water offshore basin. Southwest Petroleum University, China National Offshore Oil Corporation and Shanghai Jiao Tong University have permitted these data and results from this experiment for publication.

The individual in this manuscript has given written informed consent (as outlined in PLOS consent form) to publish these case details.

### Shear flow facility

**Figure 1:**
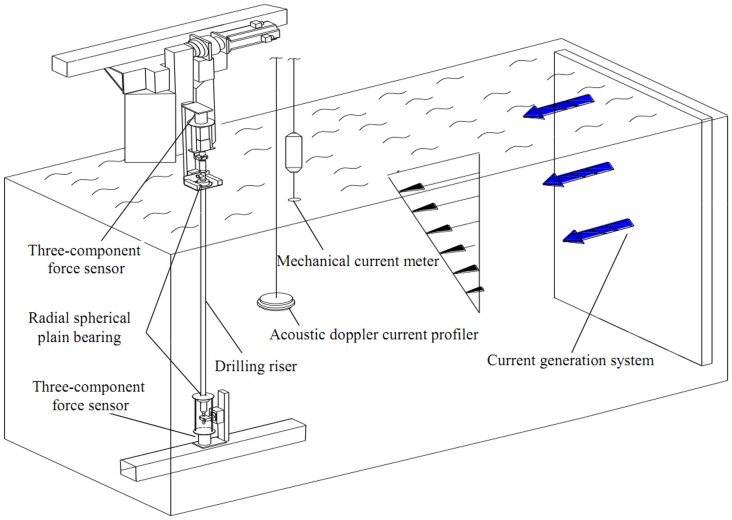
Simplified sketch of the setup.

**Figure 2:**
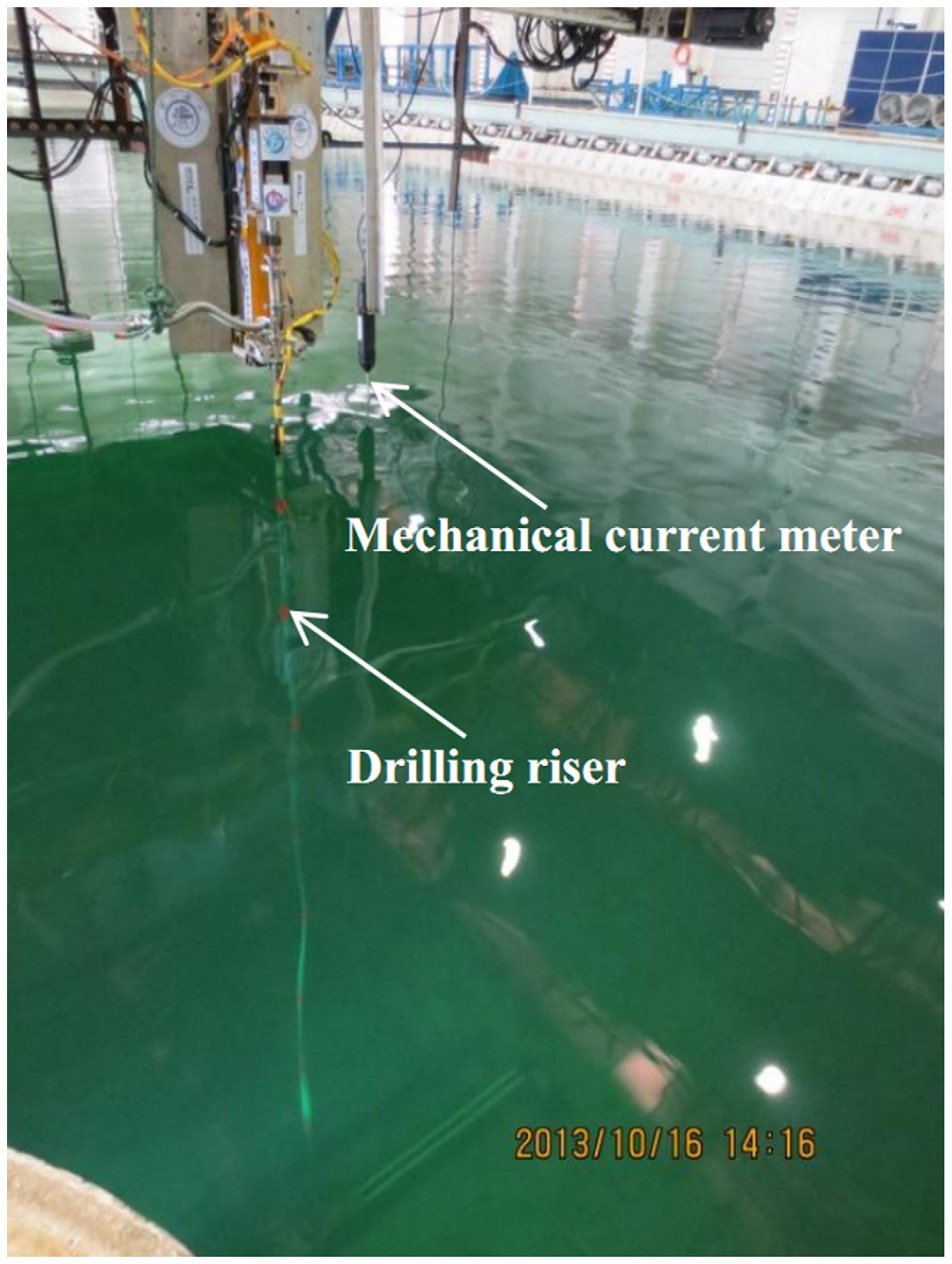
Physical experimental facility.

Experiment was conducted in a deep-water offshore basin, which was 50 m long, 40 m wide, and 10 m deep at the State Key Laboratory of Ocean Engineering. The experimental setup contained current generation system, one drilling riser model, and data acquisition system. The simplified sketch of the setup is shown in Figure 1 and the physical experimental facility is depicted in Figure 2.

**Figure 3:**
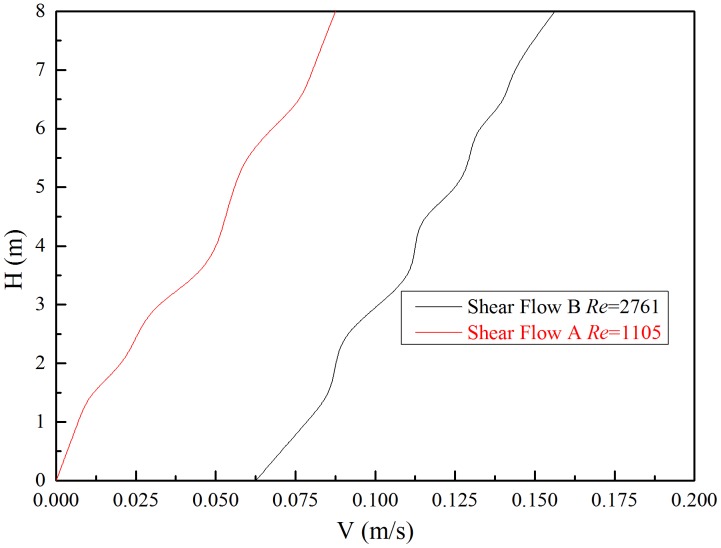
Two different shear flows generated by current generation system in the experiment.

Shear flow was generated by current generation system in our experiment. Current generation system is at the right side of the deep-water offshore basin and it can generate various types of current by controlling the parameters of the system. The current velocity profile can be measured by two current meters. One mechanical current meter was installed at the surface of the water which can measure the velocity of the surface layer of the shear flow velocity and acoustic Doppler current profiler (ADCP) was installed in the middle depth of the basin and it was used to measure the current profiler under 1 m of the water surface as shown in Figure 1. Through flow calibration, we have generated two kinds of shear flow for our tests and the shear flow profiler were measured by the two current meters as shown in Figure 3. Shear flow velocity can be expressed as follow[27]: 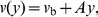
(1)where *v*_b_ is the velocity at the bottom of the basin in m/s, *y* is depth along the riser, *A* is slope of the flow profile. Shear parameter can be defined as follows[27]: 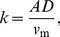
(2)where *k* is the shear parameter, *D* is the out diameter of the riser in m, *v*_m_ is the velocity at the middle of the riser in m/s.

The Reynolds number under the shear flow can be calculated as follows[27]: 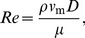
(3)where 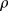 is the density of the water in kg/m^3^, *µ* is the dynamic viscosity of the water in Pa·s.

The velocity of shear flow A ranges from 0 m/s to 0.0875 m/s and the velocity of shear flow B ranges from 0.0625 m/s to 0.15625 m/s. The fitting expression of shear flow A is 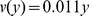, and shear flow B is *v*(*y*) = 0.0625+0.011*y*. Both the two types of the shear flows' shear parameter are 0.00626 and the *v*_m_ are 0.04375 m/s and 0.109375 m/s respectively. The corresponding Reynolds numbers are 1105 and 2761 respectively. Given this range, a fully turbulent vortex street was formed in the wake[9].

### Test drilling riser model and data acquisition

**Figure 4:**
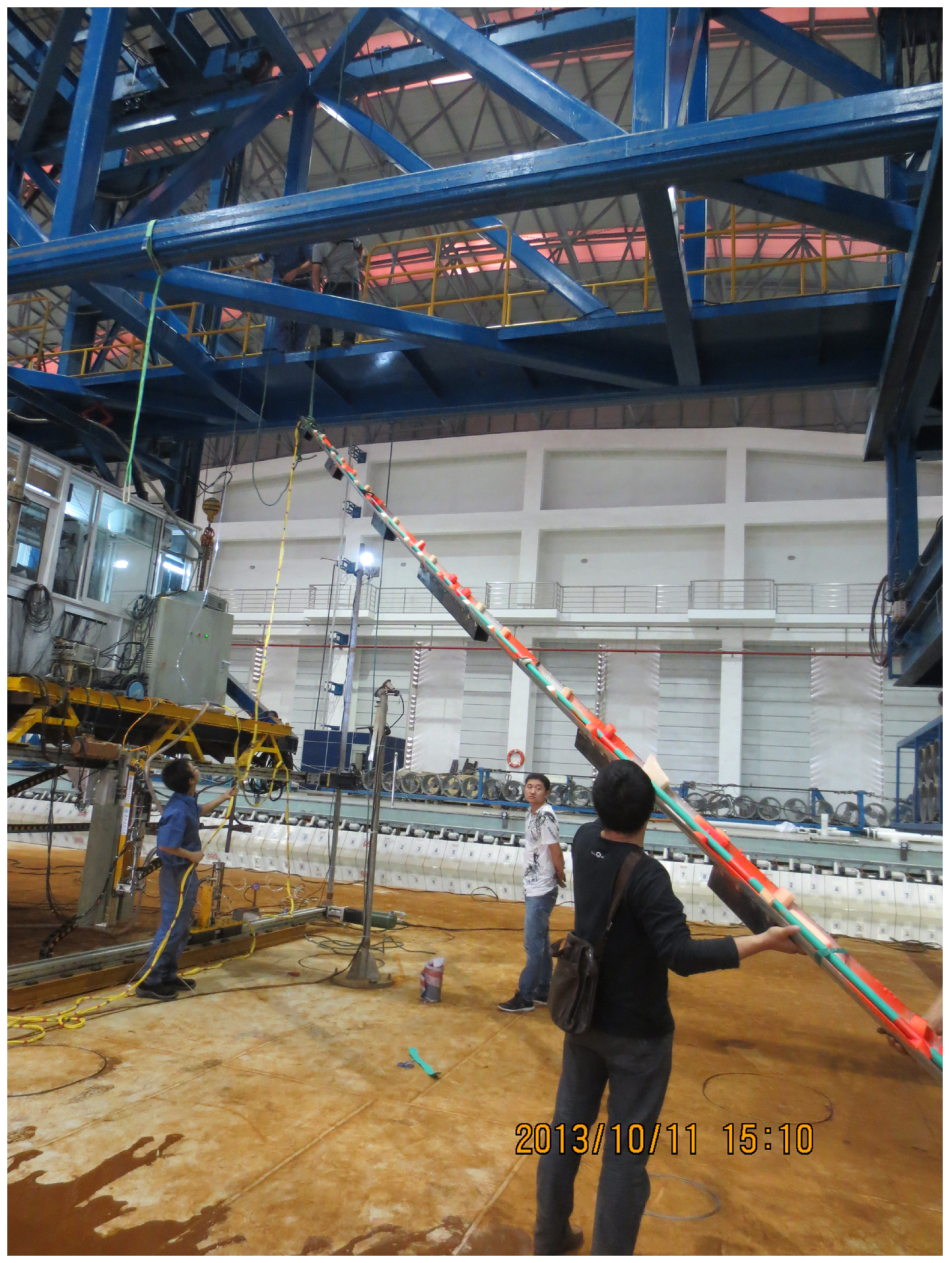
Riser model installed process.

**Figure 5:**
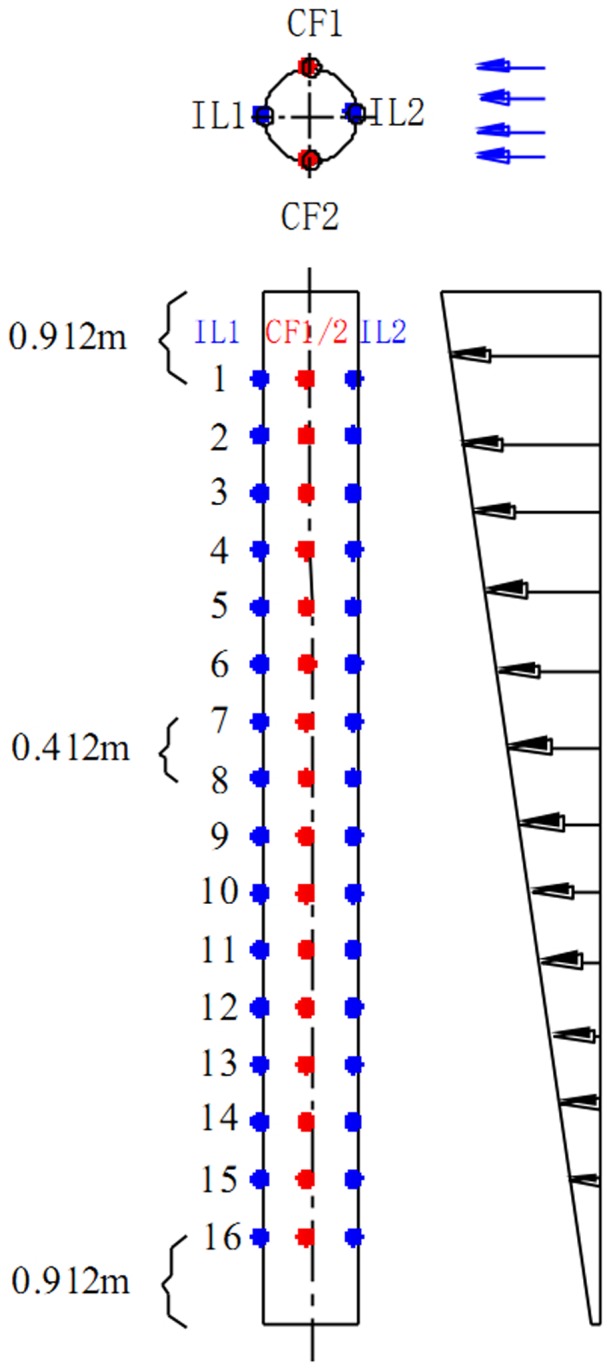
Arrangement of the fiber bragg grating sensor.

Test drilling riser model was made of PVC and its main physical properties are listed in Table 1. The riser was towed vertically by the radial spherical plain bearings at the both sides of the riser as shown in Figure 1 and the riser model installed process is depicted in Figure 4. Data acquisition mainly contained fiber grating sensor interrogating system, FBG strain sensors and three-component force sensor. FBG strain sensors were mounted along the CF and IL directions of the riser to measure VIV responses. Sixteen locations were selected to instrument FBG strain sensors, the arrangements are shown in Figure 5. “CF_1” and “CF_2” were used to capture the CF vibration, and “IL_1” and “IL_2” were used to capture the IL vibration. The sampling rate was 250 Hz. Pretension was 25 N and was exerted on drilling riser model before the beginning of the experiment. Acquisition time was more than 5 minutes after the shear flow velocity was stabilized.

**Table 1:** Main physical properties of the drilling riser model.

Item	Value
Model Length (m)	8
Thickness (m)	0.0025
Out Diameter (m)	0.025
Mass in air ( 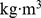 )	1570
Bending Stiffness ( 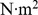 )	36
Pretenstion (N)	25
1^st^ Natural Frequency in water (Hz)	0.45
2^nd^ Natural Frequency in water (Hz)	1.18

## Data Analysis

The displacement of the riser in the CF direction or IL direction at any time can be calculated by[25]: 
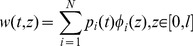
(4)where *t* denotes the time, *z* is the axial position along the riser, 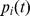 is the 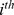 modal weight of displacement, and 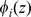 is 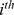 mode-shape of displacement.

The curvature then can be expressed as: 
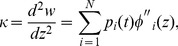
(5)where 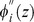 is 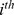 mode-shape of the curvature.

The mode-shape of displacement is sinusoidal as the riser is a beam simply supported at both ends. Therefore, 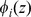 can be expressed as follows: 
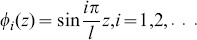
(6)


So, the curvature can be rewritten as: 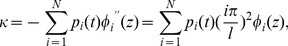
(7)


The relationship between curvature and strain is as follows: 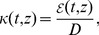
(8)where *D* is the diameter of the riser.

Combing Eq. (7) and Eq. (8), the strain can be written as: 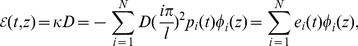
(9)

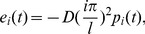
(10)where 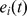 is 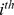 modal weight of strain with respect to 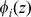.

To analyze strain amplitude without the influence of initial drag forces, static deformation generated by the latter is eliminated through the averaging method.

## Results and Discussion

The difference between uniform flow and shear flow in VIV is the irregular vortex shedding frequency along the riser under the latter. This occurrence is caused by the decrease of the shear flow velocity with the water depth. It can be inferred from the well-known Strouhal relation that the vortex shedding frequency is determined as follows: 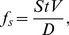
(11)where 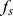 is the vortex shedding frequency in Hz; 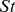 is the Strouhal number (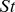 = 0.18)[2], [6]; *V* is the current speed in m/s; and *D* is the riser diameter in m. The velocity ranges from 0 m/s to 0.0875 m/s and 0.0625 m/s to 0.15625 m/s at Reynolds numbers of 1105 and 2761, respectively. From the Strouhal relation, the calculated vortex shedding frequency ranges from 0 Hz to 0.63 Hz and 0.45 Hz to 1.125 Hz at Reynolds numbers of 1105 and 2761, respectively.

**Figure 6:**
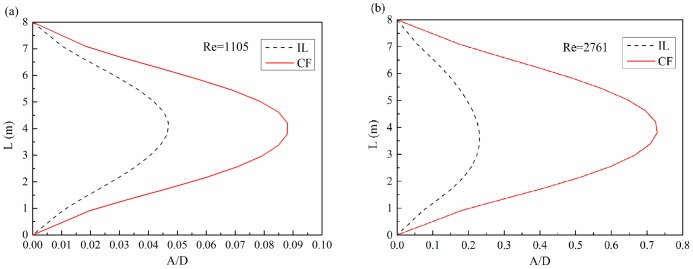
Standard deviation of displacement along riser with the Reynolds number of 1105 and 2761 under the shear flow at the same shear parameter: (a) Reynolds number of 1105; (b) Reynolds number of 2761.

Figure 6 depicts the standard deviation of displacement along the riser at the two different Reynolds numbers. The maximum velocities are 0.0875 m/s and 0.15625 m/s at Reynolds numbers of 1105 and 2761, respectively. Hence, the corresponding vortex shedding frequencies are 0.63 Hz and 1.125 Hz at the two different Reynolds numbers, respectively. The first natural frequency is 0.45 Hz, and the second natural frequency is 1.18 Hz. Therefore, Figure 6 shows that the first-order mode is the dominant vibration mode.

**Figure 7:**
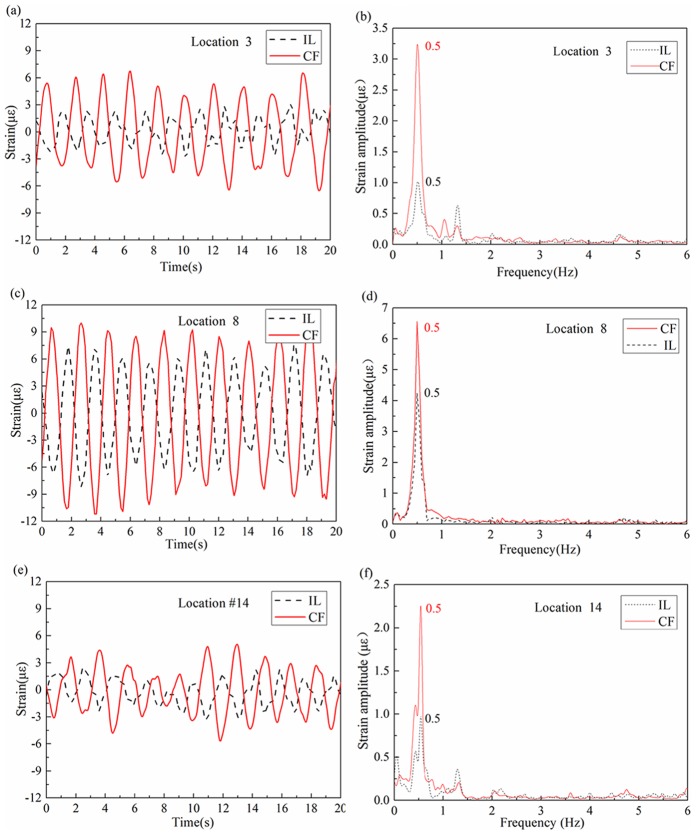
Strain response at location 3, 8 and 14 with Reynolds number of 1105 under the shear flow at the same shear parameter: (a), (c) and (e) strain time-history in the IL and CF directions; (b), (d) and (f) corresponding FFT spectrum in the IL and CF directions.

**Figure 8:**
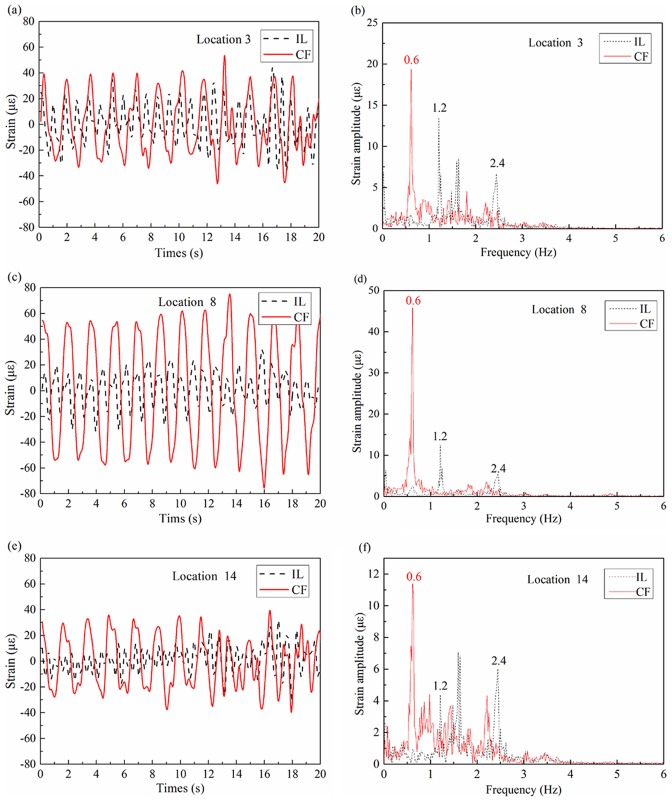
Strain response at location 3, 8 and 14 with Reynolds number of 2761 under the shear flow at the same shear parameter: (a), (c) and (e) strain time-history in the IL and CF direction; (b), (d) and (f) corresponding FFT spectrum in the IL and CF direction.

Figures 7(a), 7(c), 7(d) depicts the strain time-history in the CF and IL directions at location 3, 8, 14 with the Reynolds number of 1105, and Figures 7(b), 7(d), 7(e) are their corresponding FFT spectrum. Figures 8(a), 8(c), 8(d) shows the strain time-history in the CF and IL directions at location 3, 8, 14 with the Reynolds number of 1105, and Figures 8(b), 8(d), 8(e) are their corresponding FFT spectrums. As shown in Figures 7 and 8, the dominant frequencies are 0.5 Hz both in the CF and IL directions with the Reynolds number of 1105 and the dominant frequencies are 0.6 Hz and 1.2 Hz in the CF and IL directions with the Reynolds number of 2761 respectively.

These results indicate that the dominant frequency in the IL direction is equal to the CF dominant frequency with the Reynolds number of 1105 under shear parameter of 0.00626. This observation is different from the findings of research on uniform flow[9], [10], [25]. However, the dominant frequency in the IL direction is twice that in the CF direction with the Reynolds number increased to 2761. This frequency agrees with the results under the effect of uniform flow[6], [9]. In addition, these figures show that the dominant frequencies are consistent at different riser locations as well as different Reynolds numbers under the shear flow effect.

The shedding frequencies decreased with riser depth. These frequencies are not uniform with the shear flow velocity effect. Consequently, modal competition will occur[19]. When the shedding frequency at a certain region along the riser approaches the riser's natural frequency, the lock-in phenomenon will occur. Under this condition, the vibration will significantly increase. Subsequently, the lock-in phenomenon will dominate the riser's vibration. Thus, the dominant frequency will be the same at different locations in the same direction under shear flow.

The first-order natural frequency of the riser is 0.45 Hz. However, the CF dominant frequencies at Reynolds numbers of 1105 and 2761 are 0.5 Hz and 0.6 Hz, respectively. The difference in dominant frequency is caused by the variation of real-time frequencies in the experiment, which is influenced by the riser tension. The real-time natural frequency is determined by using the following Eq. (12)[25]: 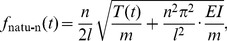
(12)where 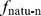 is the real-time natural frequency in Hz, 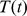 is the real-time tension of the riser in experiment in N, *l* is the length of the riser in m, *n* is the mode order, *EI* is the bending stiffness of the riser in N·m^2^, *m* is the mass per unit length in kg.

**Figure 9:**
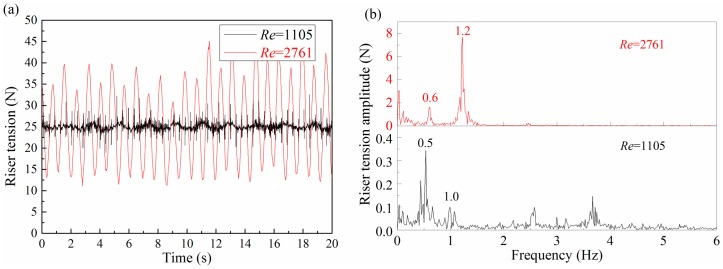
Tension response at the Reynolds number of 1105 and 2761 under the shear flow at the same shear parameter: (a) tension time-history; (b) corresponding FFT spectrum.

The riser tension periodically varied with the occurrence of the VIV. In addition, the riser tension amplitude increased with the increase of the Reynolds number at the same shear parameter, as shown in Figure 9. Thus, the dominant frequency in the experiment differs minimally from the first-order natural frequency. Eq. (12) shows that the increasing tension will increase the riser's real-time natural frequency. Hence, the real-time dominant frequency is larger than the first-order natural frequency and this frequency will increase with the increase in Reynolds number at the same shear parameter.

In addition, the dominant frequency in the IL direction is similar to that in the CF direction with a Reynolds number of 1105. Moreover, the dominant frequency in the IL direction is twice that in the CF direction with a Reynolds number of 2761. This phenomenon may be caused by the interaction effect between the IL and CF vibration and vortex shedding form. Figures 7 and 8 show that the IL and CF have several peaks and appear at each other's FFT spectrum. This indicates that there may be some kind of interaction between the IL and CF vibration. The vibration in the CF direction increases significantly under the lock-in condition. Subsequently, effect on IL vibration from the CF vibration is significantly. However, the IL vibration is clearly limited because its frequency is far from the natural frequency of the riser, and vortex shedding induced force in the IL direction is limited. Thus, the effect of vortex shedding on IL vibration in the lock-in region is extremely limited and unable to dominate the whole vibration of the riser at a low Reynolds number in shear flow. On the one hand, the vibration in the IL direction from the vortex shedding is limited to the low Reynolds number under shear flow. On the other hand, the effect on IL vibration from the CF direction is significant. Under the effects of these two aspects, the vibration in the IL direction is dominated by the interaction from the CF direction. Thus, the dominant frequency in the IL direction is the same as that in the CF direction at the Reynolds number of 1105 under shear flow in our experimental condition.

As mentioned above, the vibration in the IL direction will be affect by both of the vortex shedding and interaction from the CF direction. When the Reynolds numbers are between 1105 and 2761, the effect from the vortex shedding on the vibration of the IL direction may increase and become more significant than the effect on IL vibration from the CF direction with the increasing Reynolds numbers at the same shear parameter, and modal competition will be more obvious. Therefore, the vibration in the IL direction is gradually dominated by the vortex shedding with the Reynolds numbers increasing from 1105 to 2761 under the effect of the modal competition between the vortex shedding and interaction from the CF direction. Consequently, the dominant frequency in the IL direction may appear as multiple frequencies and vortex shedding frequency become more significantly in the vibration of the IL direction with the Reynolds numbers increasing from 1105 to 2761.

In addition, the effect of vortex shedding on IL vibration in the lock-in region increased with the increasing Reynolds number and dominated the IL direction's vibration. In the lock-in region, vortices are alternately shed from the IL direction of the riser periodically. Moreover, the riser regularly vibrates in the IL direction. While vortices are alternately shed from both sides of the CF direction, the riser vibrates in the CF direction periodically. Thus, the dominant vibration frequencies of IL are almost twice those of CF in the lock-in region[6], [9], [10]. For the lock-in region that dominates the vibration of the whole riser, the dominant vibration frequencies of IL are almost twice those of CF with increasing Reynolds number under the shear flow effect. These results are in agreement with the uniform flow effect. Peaks observed in Figure 8 also show the interaction between the IL and CF directions. However, the IL vibration from the CF vibration is much smaller than the drag force effect.

From a theoretical perspective, only one peak value should be found in the spectral figures[9]. However, several peaks were observed in the IL and CF spectral figures. Peaks become noticeable with the increase in Reynolds number. As mentioned, several peaks were due to the interaction between the IL and CF vibration. These peaks appear at each other's FFT spectrum. In addition, some peaks at high modal were observed. When the Reynolds number is 2761, 2.4 Hz peaks in the IL direction were observed. Hence, the high-order mode may affect the VIV under shear flow with increasing Reynolds number. The phenomenon is caused by the dominance of a certain mode over the VIV of the riser under the effect of shear flow. However, the vortex shedding frequency decreased along with the riser under shear flow. Shedding frequency close to the water surface is larger than that of the lock-in region because of higher velocity at the water surface. These frequencies may affect VIV in terms of the occurrence of a high-order mode. Hence, we can conclude that the multi-modal phenomenon appeared in VIV. Moreover, the multi-modal phenomenon is more apparent with an increasing Reynolds number under the shear flow effect.

The strain amplitude at location 8 is larger than that in the other two locations. Moreover, the strain amplitude in the CF direction increased significantly with the increasing Reynolds number. This finding is consistent with the corresponding standard deviation of displacement results shown in Figure 6. The phenomenon is caused by the fact that the IL dominant frequency is twice the CF dominant frequency. Hence, the IL dominant frequency is far from the one order natural frequency when lock-in occurs. Thus, the CF vibration is more significant than the IL vibration. VIV is at the one-order mode. The maximum deformation is at the middle of the riser, which determined the strain amplitude of the different location along the riser. The lock-in region increased with increasing Reynolds number at the same shear parameter. Moreover, the effect of the lock-in phenomenon on the riser is more apparent. Thus, the strain amplitude increased with the Reynolds number at the same shear parameter when VIV is at the same order mode.

Figure 9 is the corresponding riser tension response at Reynolds numbers of 1105 and 2761. Results of the riser tension response are consistent with the standard deviation of displacement and strain response results.

**Figure 10:**
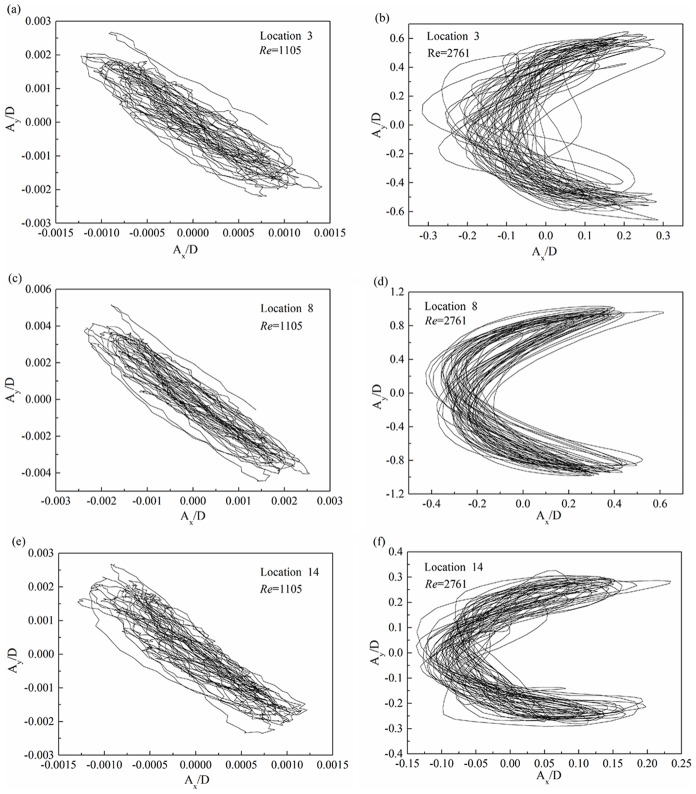
Displacement trajectories at location 3, 8 and 14 with the Reynolds number of 1105 and 2761 under the shear flow at the same shear parameter: (a), (c) and (e) Reynolds number of 1105; (b), (d) and (f) Reynolds number of 2761.

Figures 10(a), 10(c), and 10(e) show the riser displacement trajectories with the Reynolds number of 1105 under shear flow. Figures 10(b), 10(d), and 10(f) depict the riser displacement trajectories with the Reynolds number of 2761 under shear flow. Figure 10 indicates that the different locations of the riser displacement trajectories are similar at the same Reynolds number under shear flow. However, the displacement trajectories vary with different Reynolds numbers. Many studies[6], [8]-[10] have reported figure-eight displacement trajectories under the effect of the lock-in phenomenon. However, we observed diagonal displacement trajectories at the Reynolds number of 1105 and crescent-shaped displacement trajectories at the Reynolds number of 2761 in our shear flow experiment. The phenomenon is caused by the fact that the IL dominant frequency is similar to the CF dominant frequency at the Reynolds number of 1105, while the IL dominant frequency is twice the CF dominant frequency at the Reynolds number of 2761. When the IL dominant frequency is equal to the CF dominant frequency, the resultant movement of the displacement trajectories is diagonal. The IL dominant frequency is twice the CF dominant frequency with an increasing Reynolds number. Moreover, the displacement trajectories will be symmetrical in the CF direction. Williamsom[5], [6], [9] reported that the trajectory shapes are also associated with corresponding phase angles between the IL and CF directions. Under the effect of the phase angles, the equilibrium point of displacement trajectories is moved to the left. Hence, crescent–shaped displacement trajectories are observed at the Reynolds number of 2761. In summary, increasing the Reynolds number affects the dominant frequency relation between the IL and CF directions. Moreover, this increase affects the phase angles. Thus, the diagonal and crescent-shaped trajectories are observed with different Reynolds numbers under shear flow.

## Conclusions

An experimental investigation on VIV of a drilling riser under the shear flow with the same shear parameter at the two different Reynolds numbers was performed in a deep-water offshore basin. By processing the experiment data, the following conclusions can be drawn:

The corresponding maximum order mode of the natural frequency for shedding frequency is the maximum dominant vibration mode and multi-modal phenomenon is appeared in VIV under the shear flow. Moreover, multi-modal phenomenon is more apparent at the same shear parameter with an increasing Reynolds number under the shear flow effect.

The riser vibrates at the natural frequency and the dominant vibration frequency increases for the effect of the real-time tension amplitude under the shear flow. The dominant vibration frequency increases with an increasing Reynolds numbers. The IL vibration frequency is the similar with the CF vibration frequency at the Reynolds number of 1105 in our experimental condition and the IL dominant frequency is twice the CF dominant frequency with an increasing Reynolds number under shear flow.

The displacement trajectories at the different locations of the riser appear the same shape and the shape is changed at the same shear parameter with an increasing Reynolds number under the shear flow. In our experimental conditions, the diagonal displacement trajectories are observed at the low Reynolds number and the crescent-shaped displacement trajectories appear with an increasing Reynolds number under shear flow.
